# Supplementary material for: Anticholinesterase Activity of Methanolic Extract of Amorpha fruticosa Flowers and Isolation of Rotenoids and Putrescine and Spermidine Derivatives
Source: Plants (Basel). 2024 Apr 24;13(9):1181. doi: 10.3390/plants13091181 (PMC11085544; doi:10.3390/plants13091181)
Supplement: Supplementary file 1 [file plants-13-01181-s001.zip › plants-2955523-supplementary.pdf]

Supplementary Data

Anticholinesterase activity of methanolic extract of *Amorpha fruticosa* flowers and isolation of rotenoids and putrescine and spermidine derivatives

Dagmar Jankovská[1,*], Nikol Jurčová[1], Renata Kubínová[1], Jiří Václavík[1], Emil Švajdlenka[1], Anna Mascellani[2], Petr Maršík[2], Kateřina Bouzková[3] and Milan Malaník[1,*]

[1] Department of Natural Drugs, Faculty of Pharmacy, Masaryk University, Palackého třída 1946/1, 61200 Brno, Czech Republic; JURCOVA.NIKOL@seznam.cz; kubinovar@pharm.muni.cz; vaclavikj@pharm.muni.cz; svajdlenkae@pharm.muni.cz
[2] Department of Food Science, Faculty of Agrobiology, Food and Natural Resources, Czech University of Life Sciences Prague, Kamýcká 129, 16500 Prague, Czech Republic; mascellani@af.czu.cz; marsik@af.czu.cz
[3] Central European Institute of Technology (CEITEC), Masaryk University, Kamenice 5/C04, 62500 Brno, Czech Republic; kamala@mail.muni.cz
* Correspondence: jankovskad@pharm.muni.cz; malanikm@pharm.muni.cz

Table of Contents

Figure S1. Structures of the compounds **1–10** isolated from flowers of *A. fruticosa*.
Figure S2. HPLC-DAD chromatogram of the crude extract at a concentration of 5 mg/mL and typical UV spectrum of phenolamides.
Figure S3. HPLC-DAD chromatogram of the ethyl acetate fraction at a concentration of 5 mg/mL and assignment of the peaks to the corresponding compounds **1–5**.

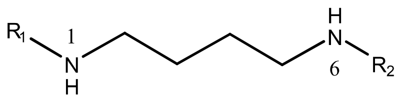

1 R_1 = A; R_2 = B
2 R_1 = A; R_2 = A

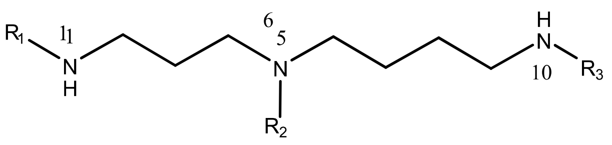

3 R_1 = A; R_2 = B; R_3 = B
4 R_1 = B; R_2 = B; R_3 = A
5 R_1 = A; R_2 = A; R_3 = A

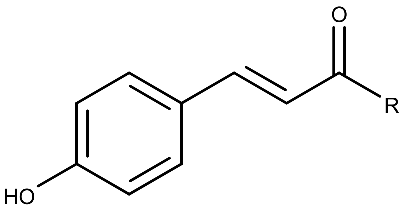

A = *trans*-*p*-coumaroyl

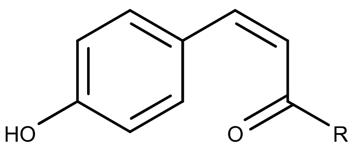

B = *cis*-*p*-coumaroyl

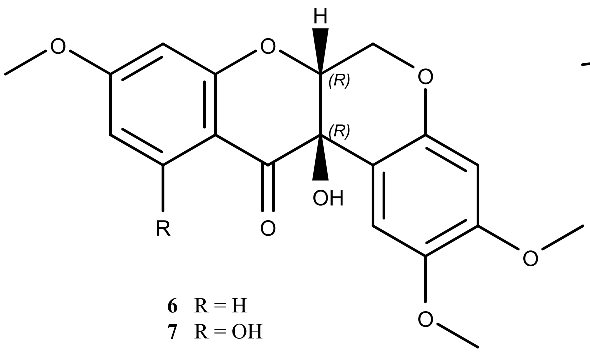

6 R = H
7 R = OH

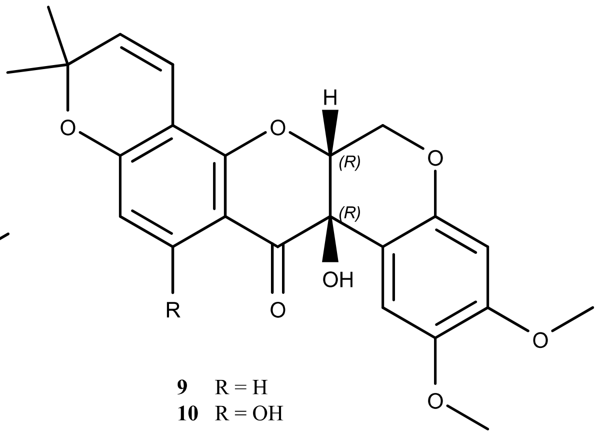

9 R = H
10 R = OH

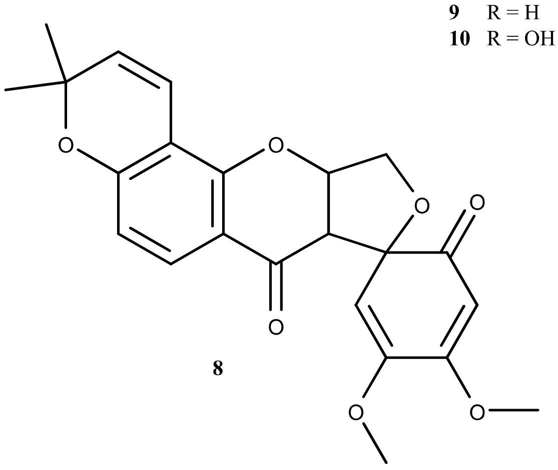

8

Figure S1. Structures of the compounds **1–10** isolated from flowers of *A. fruticosa*.

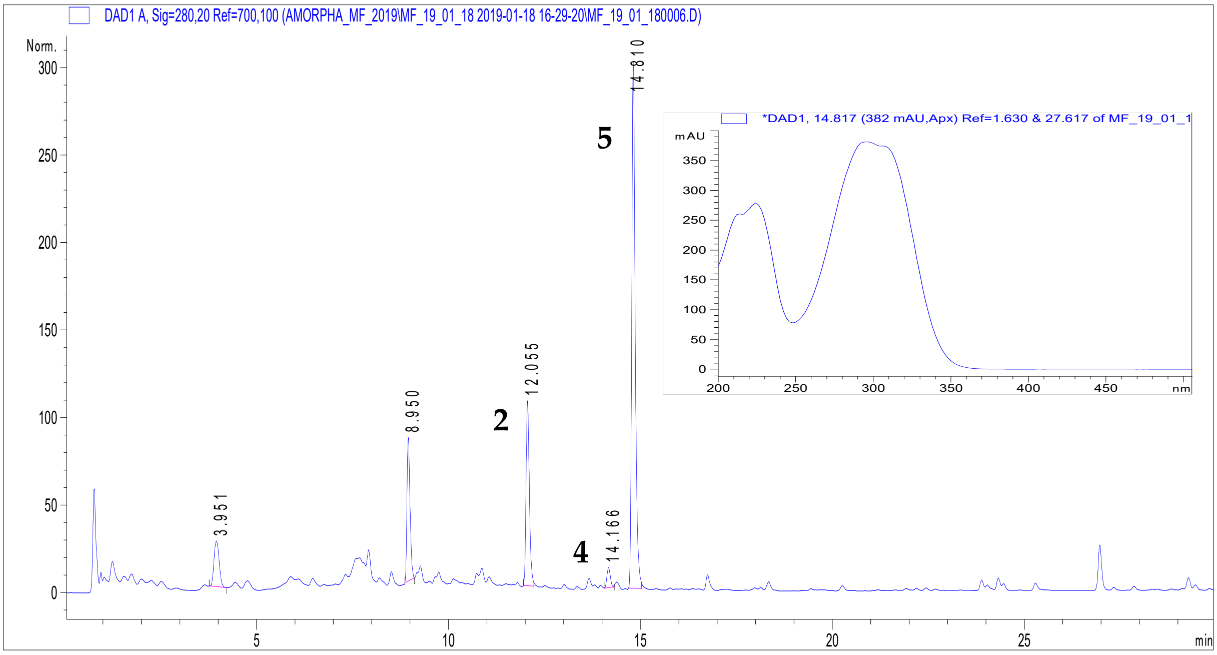

Figure S2. HPLC-DAD chromatogram of the crude extract at a concentration of 5 mg/mL and typical UV spectrum of phenolamides.

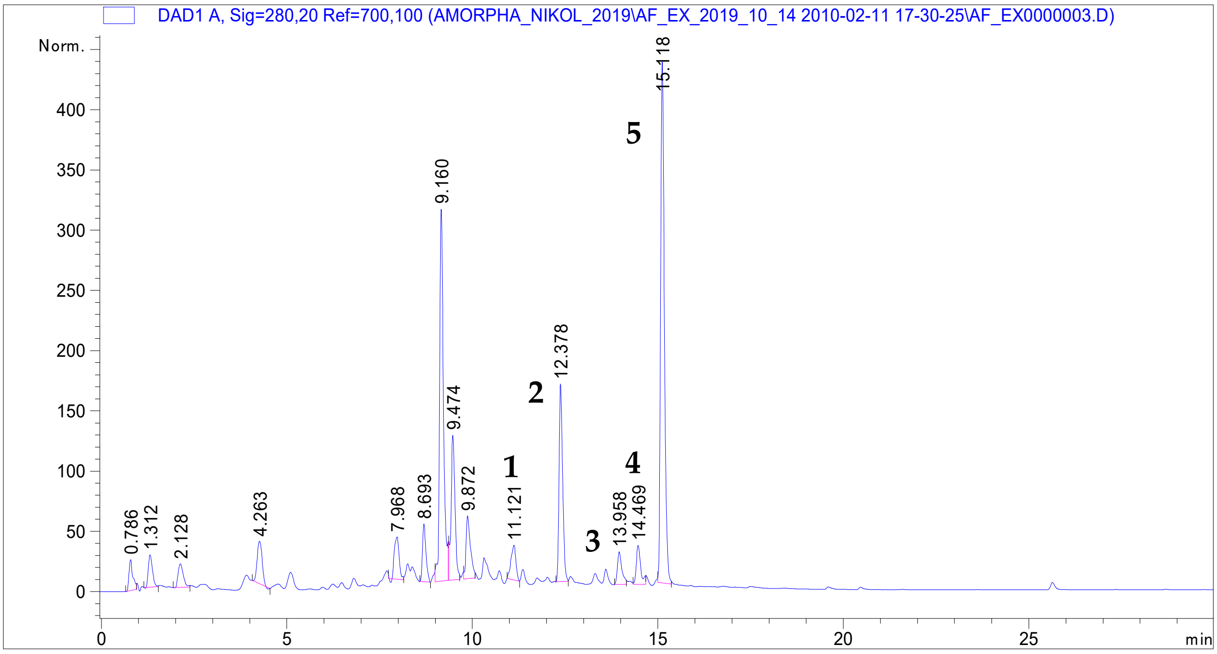

Figure S3. HPLC-DAD chromatogram of the ethyl acetate fraction at a concentration of 5 mg/mL and assignment of the peaks to the corresponding compounds **1–5**.
